# Editorial: Neural Stem Cells of the Subventricular Zone: From Neurogenesis to Glioblastoma Origin

**DOI:** 10.3389/fonc.2021.750116

**Published:** 2021-09-17

**Authors:** Esperanza R. Matarredona, Natanael Zarco, Carmen Castro, Hugo Guerrero-Cazares

**Affiliations:** ^1^Departamento de Fisiología, Facultad de Biología, Universidad de Sevilla, Seville, Spain; ^2^Neurosurgery Department, Mayo Clinic Jacksonville, Jacksonville, FL, United States; ^3^Área de Fisiología, Facultad de Medicina Universidad de Cádiz, Cádiz, Spain; ^4^Instituto de Investigación e Innovación en Biomedicina de Cádiz (INiBICA), Cádiz, Spain

**Keywords:** glioblastoma, subventricular zone, neurogenesis, cerebrospinal fluid, glioma stem cells

Glioblastoma (GBM) is the most common and aggressive primary tumour of the adult central nervous system. Patients with a GBM diagnosis present a poor prognosis and median survival of less than 2 years. Despite the use of standard of care, which includes surgery, chemotherapy and radiation, recurrence is almost inevitable. Understanding the cellular and molecular cues that underlie the origin and development of this aggressive tumour, may lead to the identification of new therapeutic targets to fight GBM progression and recurrence. The Research Topic “Neural stem cells of the subventricular zone: from neurogenesis to glioblastoma origin” includes 13 articles which provide a general view on the influence of the subventricular zone (SVZ), a region that contains neural stem cells (NSCs) in the adult brain, in the origin and progression of GBM (**Figure 1**).

**Figure 1:**
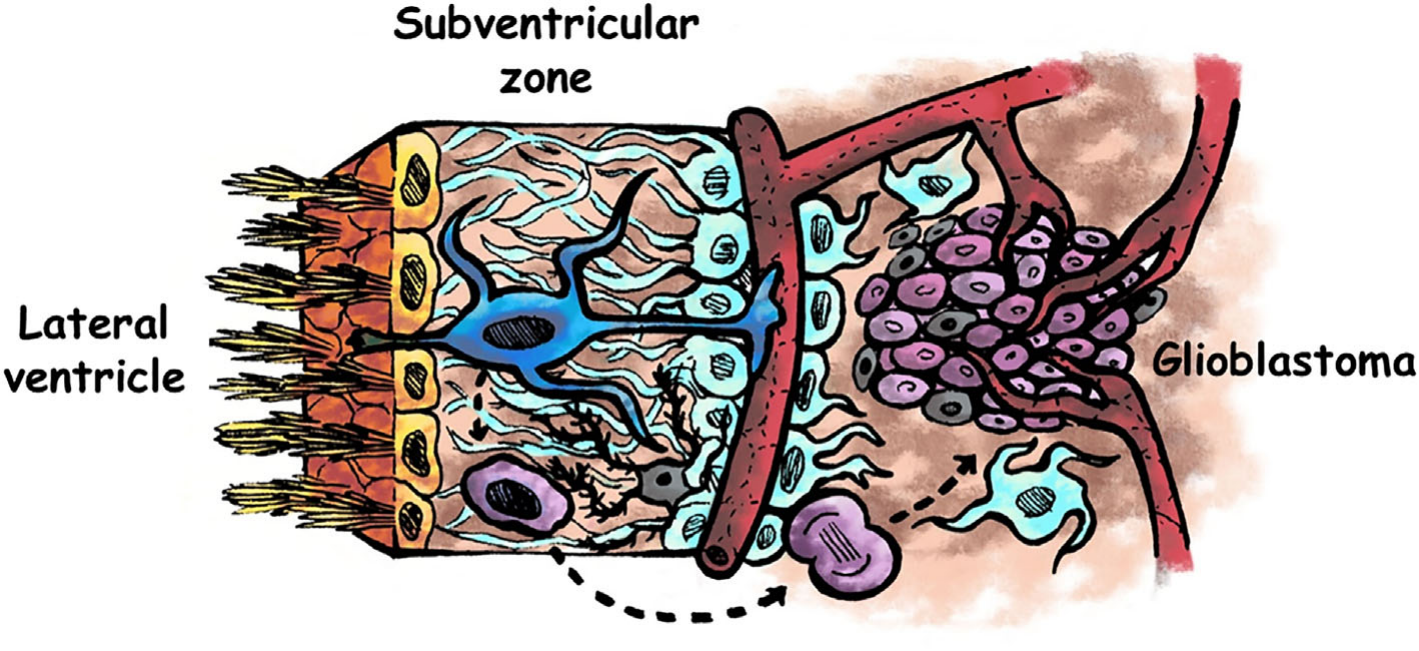
Schematic representation of the human adult subventricular zone and an adjacent glioblastoma. Ependymal cells are represented in yellow, neural stem cells in blue, astrocytes in pale blue, microglia in grey, glioma stem cells in purple, and other type of glioma cells in pale purple. Neural stem cells of the subventricular zone may acquire driver mutations that generate glioma stem cells, which divide to form the tumour mass. Factors present in the cerebrospinal fluid flowing the lateral ventricles may intervene in the genesis and/or in the progression of glioblastomas. Modified from (1).

Extremely invasive, GBMs cannot be completely resected by surgery, and are resistant to radiation and chemotherapy. A subpopulation of cells called glioma stem cells (GSCs), which exhibit NSC properties, are responsible for drug resistance and tumour recurrence. A couple of articles in this Topic analyse similarities and differences between NSCs of the SVZ and GSCs. Lombard et al. describe some features shared by GSCs and NSCs and show that the SVZ is a preferred destination site for GSCs, probably due to chemoattractive cues released within the SVZ. The authors summarize some studies showing the role of the chemokine CXCL12 and the protein pleiotrophin as key players in the migration of GSCs from the tumour mass towards the SVZ. In addition, they discuss possible reasons by which GSCs nested in the SVZ benefit from a supportive environment and present an increased resistance to radiation and chemotherapy. In line with this work, Bakhshinyan et al. review different mechanisms involved in the intrinsic and extrinsic regulation of SVZ-derived NSCs that are shared by GSCs. Their analysis shows that the dysregulation of these mechanisms may induce aberrant growth signals, increased invasiveness, sustained angiogenesis or evasion of apoptotic death. The analysis of the differences between NSC and GSC regulatory mechanisms may offer new avenues for the generation of novel targeted therapies for GBM. Noteworthy, NSCs residing in the other neurogenic niche of the adult mammalian brain, the hippocampus, have not been involved in gliomagenesis. Fontán-Lozano et al. provide possible explanations for this fact by analysing cellular and molecular differences between the SVZ and the hippocampal niches, as well as genotypic and phenotypic characteristics of the NSCs present in both niches that might confer SVZ NSCs more opportunities to become tumorigenic than hippocampal NSCs. One of the main differences between these two neurogenic niches is the direct contact with the cerebrospinal fluid (CSF), existing in the SVZ but not in the hippocampus. Thus, factors present in the CSF might intervene in NSC biology and in GBM development (**Figure 1**). Indeed, an interesting research article published in this Topic by Carrano et al. investigates the effect of human CSF on primary-cultured GSCs. They show that CSF derived from GBM patients induces an increase in proliferation and migration of GSCs *in vitro*. The transcriptome analysis of GBM cells exposed to CSF reveals alterations in gene expression in pathways promoting cell malignancy. In addition, the authors test these effects *in vivo*, by injecting GBM cells encapsulated in a hydrogel containing human CSF and demonstrate that animals receiving this combination generate larger and more proliferative tumours than controls. These reported effects of CSF on GBM malignancy could be partially responsible for the observed increased aggressiveness of those tumours close to the SVZ. The relevance of the proximity to SVZ in GBM malignancy has also been analysed by some other authors in this Topic. Ripari et al., in an interesting experimental approach, show that animals that receive GBM engraftments close to the lateral ventricles (and therefore to the SVZ) develop larger and more proliferative tumours than animals with GBM grafts distal to the lateral ventricles. GBM proximity to lateral ventricles also leads to decreased median survival of the animals. A noticeable aspect of this study is that the authors also analyse the influence of GBM proximity on the SVZ population of NSCs and progenitors. They demonstrate that tumour proximity to the lateral ventricles induces a decrease in the proliferation of SVZ NSCs and their progeny. These results emphasize the importance of deciphering bidirectional molecular signalling between GBM and the SVZ to identify pathways contributing to tumour progression in patients with GBM located proximal to the SVZ. In relation to this, the article by Mistry et al. analyses possible correlations between patient survival and GBM distance to the SVZ and demonstrates a significantly decreased survival when the tumour contacted the SVZ. However, they do not report a survival correlation with the GBM-SVZ distance. Their results have clinical relevance to test differential effectiveness of SVZ radiation in patients with SVZ-contacting or non-contacting GBMs. Other predictive factors for GBM progression that may help to make more personalized and precise treatments have been studied by Jiang et al. They show that SVZ involvement is correlated with higher risk for non-local progression in patients with IDH-wildtype GBM. This correlation is also described for male gender and MGMT promoter methylation.

Experimental models of GBM are crucial to understand the mechanisms involved in the progression of this devastating disease and to find more efficient treatments. Besides, as IDH-wildtype and IDH-mutant GBMs differ in their cell of origin and in their genetic alterations, different animal models need to be designed for both GBM types. Gomez-Oliva et al. have carefully evaluated the different experimental models used in the study of GBM. They first describe advantages and disadvantages of classical approaches such as cell cultures from GBM cell lines or patient-derived cells and xenografts to continue with more novel approaches such as genetically engineered mouse models, organotypic cultures, brain organoids or 3D-bioprinted mini-brains. Kim et al. give an overview of genetic alterations and cell-of-origin in IDH-wildtype and IDH-mutant GBMs and discuss recent genetically engineered mouse models in which NSCs or progenitor cells are transformed by specific genetic alterations to model either IDH-wildtype or IDH-mutant GBM.

There is consensus that IDH-wild type GBM may arise from accumulation of somatic mutations in SVZ NSCs and/or in glial precursor cells that confer growth advantages resulting in uncontrolled proliferation. These driver mutations could originate from genetic alterations as well as by epigenetic modifications. Two articles of this Topic analyse this possibility. Lozano-Ureña et al., based on previous reports describing a dysregulation of the imprinting pattern in different tumours, show that there is an extensive alteration in the expression of imprinted genes in GBMs. Furthermore, they demonstrate that adult NSCs from the human SVZ cannot be distinguished from GBM cells based on imprinted gene expression data, which supports the hypothesis that NSCs are the cells-of-origin of IDH-wild type GBMs. Valor and Hervás-Corpión review multiple epigenetic activities that are involved in glioma malignancy and some therapeutic approaches proposed to overcome these epigenetic changes. For instance, they describe in detail the role of Polycomb repressive complexes or the histone variant H3.3 in the maintenance of the GSC phenotype.

This Research Topic also includes some articles providing potential therapeutic strategies for GBM. Rackov et al. analyse the effect of Nilo1, a monoclonal antibody that marks NSCs and early progenitors, on patient-derived GSCs. They show that Nilo1 recognizes GSCs and reduces cell viability and self-renewal in a subset of GSCs. Their results open the possibility of studying the effect of this antibody-based therapy in preclinical studies alone or in combination with other drugs. Another therapeutic option for GBM has been discussed by Geribaldi-Doldán et al. focusing on one important component of both SVZ and GBM niches: the microglia. Microglial cells within the GBM microenvironment acquire a tumour-supportive phenotype, which is analysed by the authors providing details on some relevant molecules and epigenetic mechanisms involved in its acquisition. Accordingly, they discuss possible therapies based on microglia as a target to complement the currently used treatments for this disease. Also, the already mentioned articles by Lombard et al. and Bakhshinyan et al., include interesting analyses on the SVZ as a potential therapeutic target in GBM. Additionally, research discussed in the articles by Carrano et al. and Ripari et al. provide evidence of the existence of factors present in the CSF and/or in the SVZ that promote GBM malignancy, which encourages the study of the identification of molecules responsible for these effects with the goal of their use as biomarkers and/or targets for this disease.

Overall, the collection of articles contained in this Research Topic contributes to the understanding of GBM from different perspectives. Firstly, it provides an analysis of the similarities and differences between GSCs and NSCs of two adult neurogenic niches: the SVZ and the hippocampus. Secondly, it highlights the role of the SVZ in GBM tumour progression and patient survival, identifying molecules in the CSF as responsible for tumour progression, aggressiveness and malignancy. Finally, it discusses the most adequate models to study GBM, and to end with, it suggests new therapeutic targets providing potential strategies for the treatment of GBM.

## Author Contributions

EM, NZ, CC, and HG-C contributed equally to this Research Topic by acting as Guest Editors and writing the editorial. All authors contributed to the article and approved the submitted version.

## Funding

EM is funded by VI Plan Propio de Investigación (Universidad de Sevilla) Grant number 2020/0000081. NZ and HG-C are funded by the NIH-NINDS K01NS110930-03 and the Neuro-oncology convergence. CC is funded by the Integrated Territorial Investment Operational Programme of the European Commission and by the Department of Department of Health and Families (Consejería de Salud y Familias) of the Regional Government of Andalusia. Project reference: ITI-0042-2019: ITI Cadiz 2019.

## Conflict of Interest

The authors declare that the research was conducted in the absence of any commercial or financial relationships that could be construed as a potential conflict of interest.

## Publisher’s Note

All claims expressed in this article are solely those of the authors and do not necessarily represent those of their affiliated organizations, or those of the publisher, the editors and the reviewers. Any product that may be evaluated in this article, or claim that may be made by its manufacturer, is not guaranteed or endorsed by the publisher.
